# Detection of Guanine and Adenine Using an Aminated Reduced Graphene Oxide Functional Membrane-Modified Glassy Carbon Electrode

**DOI:** 10.3390/s17071652

**Published:** 2017-07-18

**Authors:** Di Li, Xiao-Lu Yang, Bao-Lin Xiao, Fang-Yong Geng, Jun Hong, Nader Sheibani, Ali Akbar Moosavi-Movahedi

**Affiliations:** 1 School of Life Sciences, Henan University, JinMing Road, Kaifeng 475000, China; lidi0722@hotmail.com (D.L.); xiaoluy1989@sohu.com (X.-L.Y.); arixxl@163.com (B.-L.X.); gengfangyong@hotmail.com (F.-Y.G.); 2 Institute of Biotechnology, Henan University, Kaifeng 475000, China; 3 Department of Ophthalmology and Visual Sciences and Biomedical Engineering, University of Wisconsin School of Medicine and Public Health, Madison, WI 53726, USA; nsheibanikar@wisc.edu; 4 Institute of Biochemistry and Biophysics, University of Tehran, Enquelab Avenue, Tehran 13145-1384, Iran

**Keywords:** aminated reduced graphene oxide, adenine, guanine, electrochemical detection, glassy carbon electrode

## Abstract

A new electrochemical sensor based on a Nafion, aminated reduced graphene oxide and chitosan functional membrane-modified glassy carbon electrode was proposed for the simultaneous detection of adenine and guanine. Fourier transform-infrared spectrometry (FTIR), transmission electron microscopy (TEM), and electrochemical methods were utilized for the additional characterization of the membrane materials. The prepared electrode was utilized for the detection of guanine (G) and adenine (A). The anodic peak currents to G and A were linear in the concentrations ranging from 0.1 to 120 μM and 0.2 to 110 μM, respectively. The detection limits were found to be 0.1 μM and 0.2 μM, respectively. Moreover, the modified electrode could also be used to determine G and A in calf thymus DNA.

## 1. Introduction

Adenine (A) and guanine (G) are vital constituents of deoxyribonucleic acids. They are very important in storing genetic information. Measuring the levels of A and G is important in bioscience and clinical diagnosis, because their quantities can act as important indicators for the diagnosis of various illnesses [1,2,3,4,5].

Numerous technical means are used for such analyses, including chemiluminescence [6], isotope dilution mass spectrometry [7], HPLC [8,9], capillary zone electrophoresis [10], and calorimetry [11]. Among these, the electrochemical methods have many merits compared with the traditional methods including real-time application, high sensitivity, fast response, and low cost [12,13]. However, the analytical sensitivities are usually very low, due to the irreversible adsorption and weak direct electron transfer capacity for both A and G on the surface of conventional electrodes.

In order to overcome the aforementioned shortcomings, many materials are used to modify electrodes, including the carboxylation of multi-walled carbon nanotubes [14], mesoporous carbon [15], and TiO_2_ nanobelts [16]. Recently, different functional membranes, such as graphene–ionic liquid–chitosan composites [17], graphene–Nafion composite membranes [18], pyridinedicarboxylic acid-graphene composite films [19], ionic liquids coated nanocrystalline zeolite materials [20], ruthenium hexachloroplatinate (RuPtCl_6_) inorganic membranes [21], titanium dioxide nanofibers-graphene oxide nanosheets nanocomposites [22], multi-walled carbon nanotubes-Fe_3_O_4_-polydopamine-Ag nanocomposites [23], carboxymethyl cellulose-halloysite nanotubular-carboxyl-functionalized multi-carbon nanotubes composites [24], and bimemtallic nanoparticles [25,26], are becoming more favorable as they achieve better electrochemical properties.

Recently, graphene oxide, nanofibers, fullerenes, carbon nanotubes, and other carbon allotropes have become important research objects for the improvement of electrochemical biosensing systems. Among them, graphene oxide (GO), combined with its finite dimension and unique structure, has demonstrated various properties such as superior mechanical flexibility, stability, and electrochemical conductivity. These unique properties make it a promising candidate for the fabrication of biosensors used for the detection of desired biomolecules [27,28]. Reduced graphene oxide (RGO) is the chemically reduced form of GO. RGOs possess improved electrical conductivity, and could be applied for the functionalization of desired biomolecules [29,30,31,32]. The main problem now is how to make an evenly dispersed graphene derivative that exhibits its proper performance in the fabrication process for a biosensor.

**Scheme 1:**
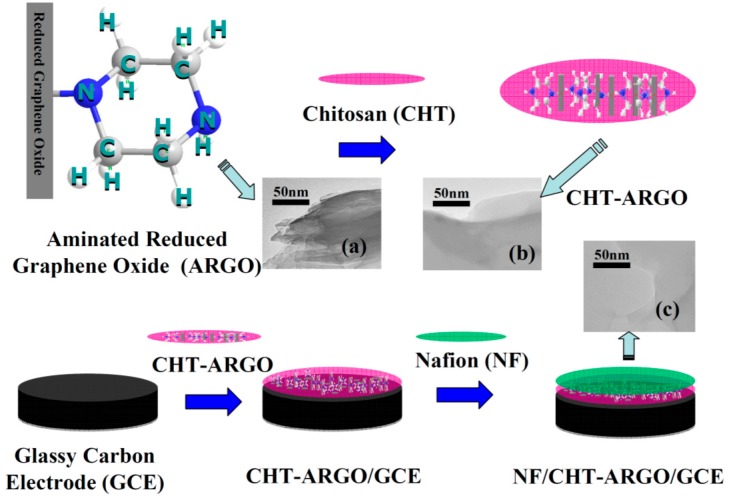
The preparation processes of NF/CHT-ARGO/GCE and the TEM images of ARGO (**a**), CHT-ARGO (**b**), and NF-CHT-ARGO (**c**). NF: Nafion, CHT: chitosan, ARGO: aminated reduced graphene oxide.

In the present study, a new electrochemical sensor based on Nafion (NF), aminated reduced graphene oxide (ARGO) and chitosan (CHT) functional membrane was proposed for the simultaneous detection of A and G. ARGO is a reduced graphene oxide covalently linked with piperazine, and is homogeneously dispersed in the membrane. CHT would be helpful to disperse the ARGO in the membrane, increase the surface area, and improve the electrochemical properties for CHT-ARGO compared with ARGO alone. The negatively charged NF may adsorb more positively charged guanine and adenine molecules to enhance the oxidation signals. Finally, the functional membrane-modified glassy carbon electrode (GCE) showed high electro-catalytic properties for the measurement of G and A (Scheme 1).

## 2. Materials and Methods 

### 2.1. Reagents

CHT (0.5% in water), Adenine (A), Guanine (G), Cytosine (C), Thymine (T), NF (5% in a mixture of alcohols (methanol, ethanol, isopropanol) and water), disodium hydrogen phosphate (Na_2_HPO_4_), sodium dihydrogen phosphate (NaH_2_PO_4_), and calf thymus DNA were purchased from Sigma (Saint Louis, MO, USA). Aminated reduced graphene oxide (ARGO) was from Shenzhen Nanotech Port Ltd. Co. (Shenzhen, China). All other chemicals were of analytical grade. All solutions were prepared in double-distilled deionized water.

### 2.2. Fabrication of NF/CHT-ARGO Modified GCE

The procedure for the preparation of the GCE was as previously described [33,34,35]. The GCE was mechanically burnished with alumina (particle sizes: 1 μm, 0.3 μm, and 0.05 μm, respectively). After a electrochemical treatment in 0.2 M sulfuric acid, the GCE was inserted in a phosphate buffer solution (PBS, 50 mM, pH 7.0), and was treated at 1.7 V for 4 min. Then, the GCE was cleaned with water and dried with dry N_2_ at 25 °C. Afterwards, 3 μL of the equal volume mixture of CHT and ARGO (4 mg/mL) and 2 μL NF were successively added to the electrode surface and dried, respectively.

### 2.3. Instruments and Detections

An electrochemical workstation (CHI650C, CH Instrument, Austin, TX, USA) was used for electrochemical investigations. A Pt wire, an Ag/AgCl-saturated KCl, and a GCE 3 mm in diameter (CH Instrument, Austin, TX, USA) were used as counter, reference, and working electrodes, respectively. The electrochemical measurements were performed in N_2_-saturated PBS (phosphate buffer solution, 50 mM pH 7.0) at 25 ± 1 °C. 

Fourier transform-infrared spectroscopy was recorded on a Spectrum Two Fourier transform infrared spectrometer (FTIR) (PerkinElmer, Beaconsfield, UK) with a KBr plate.

Electron micrograph images of the samples were evaluated using a TEM (JEM-1400, JEOL, Tokyo, Japan) operating at 80 kV.

### 2.4. Preparationof the Calf Thymus DNA Sample 

The quantity of G and A was determined for the calf thymus DNA hydrolysates [17]. First, 10 mg of the calf thymus DNA was digested with 1 M HCl (3 mL) in a sealed glass bottle. Then, the sample was incubated at 90 °C for approximately 80 min. After adjusting the pH value to 7.0 with 1 M NaOH, 5 mL of the prepared solution was injected to the electrochemical cell for further electrochemical analysis.

### 2.5. HPLC Analysis on the Real Sample

These analyses were carried out on an HPLC instrument (Waters 1515, Parsippany, NJ, USA) equipped with a UV/Visible Detector Waters 2489. A C18 HPLC column (4.6 × 250 mm, 5 μm particle size) was used as a stationary phase. Sample peaks were analyzed using the Breeze software. The mobile phase was a mixture of methanol 4%-sodium acetate 0.6 M for isocratic elution. All solutions, including the ATGC (four bases) mixed standard samples and calf thymus DNA samples were filtered (0.22 μm filter) before use. The filtered samples (20 μL) were injected into the chromatographic column for analysis. The analysis temperature was maintained at 30 °C, the flow rate was kept constant at 1.0 mL/min, and the wavelength was set at 260 nm for detection [10,11,36].

## 3. Results

### 3.1. Microscopic and Structural Studies

The TEM images of ARGO, CHT-ARGO, and NF-CHT-ARGO are shown in Scheme 1a–c, respectively. The ARGO was homogeneously dispersed in CHT (b) and NF-CHT (c), which could help maintaining a larger surface area on the GCE. The effective surface area was significantly increased after being modified with CHT-ARGO compared with ARGO.

**Figure 1:**
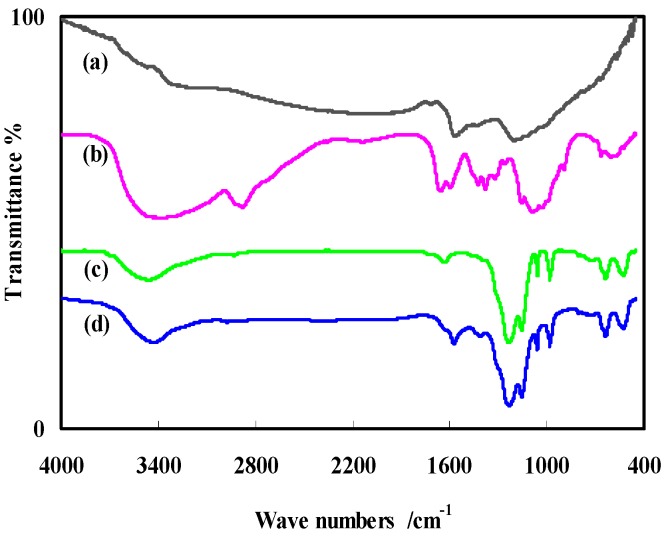
FTIR spectra of ARGO (curve a), CHT (curve b), NF (curve c), and the mixture of NF-CHT-ARGO (curve d).

The FTIR spectra of ARGO, CHT, NF, and NF-CHT-ARGO are shown in Figure 1 (curves a, b, c, and d, respectively). ARGO exhibited a group of characteristic peaks at around 3203 cm^−1^, 1550 cm^−1^, 1432 cm^−1^, and 1006 cm^−1^. The presence of the amino groups was evident by the weak peaks at 1432 cm^−1^ and 1550 cm^−1^ due to N–H bending (curve a). The abundance of –OH and –NH_2_ groups, around 3425 cm^−1^, 1072 cm^−1^ due to O–H and 1650 cm^−1^, 1591 cm^−1^ due to N–H, makes CHT (curve b) a prefect biocompatible material. The characteristic peaks of the –SO_3_^−^ group of NF at around 1220 cm^−1^ and 1150 cm^−1^ appeared in both NF (curve c) and NF-CHT-ARGO (curve d) [17,31,37].

### 3.2. Characterization of Electrochemical Behavior of Different Modified GCEs

**Figure 2:**
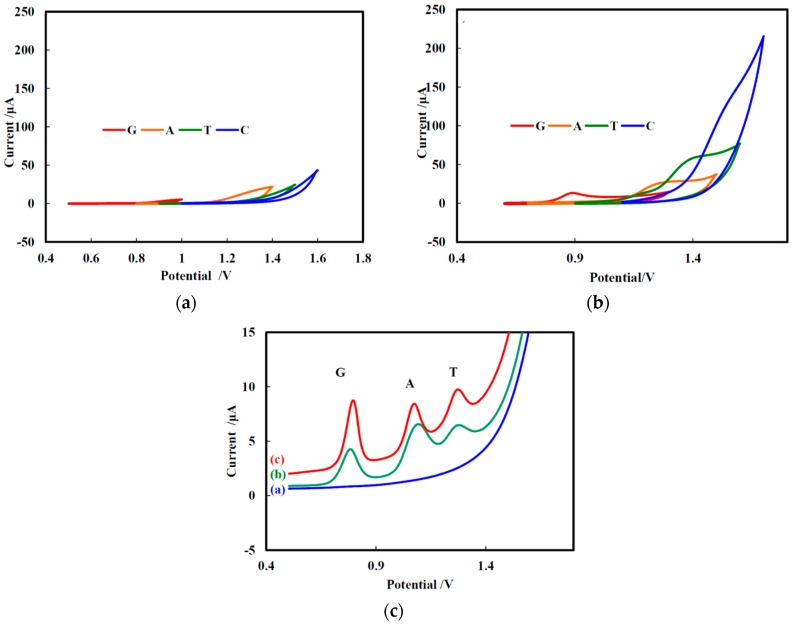
Cyclic voltammograms (CVs) of G, A, T, and C (each 1 mM) at (**a**) bare GCE and (**b**) NF/CHT-ARGO/GCE in buffer solution (pH 7.0) at a scan rate of 50 mV/s; (**c**) Differential pulse voltammograms (DPVs) of G, A, and T at bare GCE (curve a), NF/ARGO/GCE (curve b), and NF/CHT-ARGO/GCE (curve c), respectively, in pH 7.0 PBS at a scan rate of 50 mV/s.

The cyclic voltammograms (CVs) of G, A, T, and C (each 1 mM) at (a) bare GCE and (b) NF/CHT-ARGO/GCE are shown in Figure 2. The CVs of bare GCE looked no redox peak was observed in Figure 2a. The G, A, and T were oxidized on NF/CHT-ARGO/GCE at 0.903 V, 1.247 V, or 1.407 V, respectively (Figure 2b). The oxidation peak currents were higher for NF/CHT-ARGO/GCE than that for NF/CHT-GO/GCE or NF/CHT-RGO (data not shown). ARGO may be more suitable for the preparation of functional membrane to detect the A and G than GO or RGO. The negatively charged NF contributes to enhance signals probably ionic interaction with the positively charged A and G molecules. A slightly smaller signal was observed for T, while the C oxidation was hardly observed (Figure 2b).

The electrochemistry of G, A, or T (each 1 mM) at various decorated GCEs were also investigated by differential pulse voltammetry (DPV), respectively, in 50 mM PBS, pH 7.0 (Figure 2c). No redox peak was observed in the range from 0.5 to 1.5 V at bare GCE (curve a). The NF/CHT-ARGO/GCE (curve c) gave three stronger oxidation peaks compared with NF/ARGO/GCE (curve b) at around 0.804 V, 1.084 V, and 1.288 V for G, A, and T, respectively. The peak-to-peak separation between G and A was 0.280 V. The separation between A and T was 0.208 V. These results may be ascribed to the high conductivity, high electrochemical activity, and the synergistic effect of the electro catalytic property of NF, CHT, and ARGO. It should be pointed out that the NF/CHT-ARGO functional membrane-decorated GCE can not only distinguish between G, A and T, but also can improve the measurement sensitivity. Though the peak-to-peak spacing between G and A (or between A and T) was 0.280 V (or 0.208 V), which was large enough to measure G, A, and T simultaneously, it may be more suitable to detect G and A on the NF/CHT-ARGO/GCE, to improve the anti-interference ability of the electrode.

### 3.3. pH Effects Values on Determination of G and A

**Figure 3:**
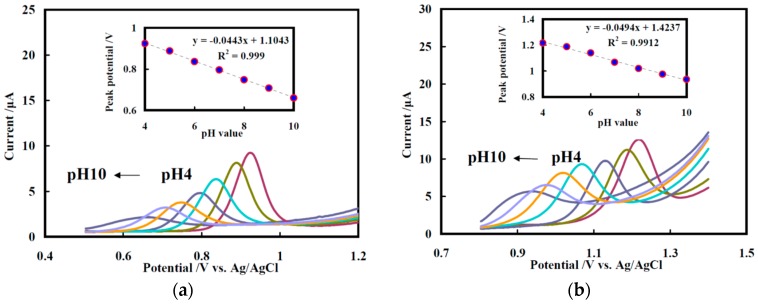
Effects of pH value on the electrochemical oxidation of 100 μM G (**a**) and 100 μM A (**b**), respectively, on NF/CHI-ARGO/GCE (accumulation time: 200 s). Insets: plots of the oxidation peaks potential versus pH value.

The pH effects on the individual oxidation of G and A, which were studied by DPVs of NF/CHT-ARGO/GCE in the pH value range from 4 to 10 (from right to left), are shown in Figure 3a,b, respectively. The oxidation peak potentials of G and A shifted positively with the decrease in pH values (from 4 to 10), indicating that the electrochemical oxidation of G or A was associated with a proton-transfer process. The relationships of the peak potentials of G and A were linear and proportional to pH values. The slopes were 44.3 mV/pH (Figure 3a, inset) and 49.4 mV/pH (Figure 3b, inset), respectively, which means that the numbers of electrons and protons involved in the mechanisms of guanine and adenine might be the same [17]. The electrochemical oxidation of G and A on NF/CHT-ARGO/GCE should be a two-electron and two-proton process, which is similar to that on graphene–NF/GCE [18]. The oxidation peak currents of G and A decreased with the increasing pH value (Figure 3). In consideration of the separation effects and measurement sensitivity, pH 7.0 was chosen as the optimum value in the later investigations.

### 3.4. Determination of G and A Using NF/CHT-ARGO/GCE

**Figure 4:**
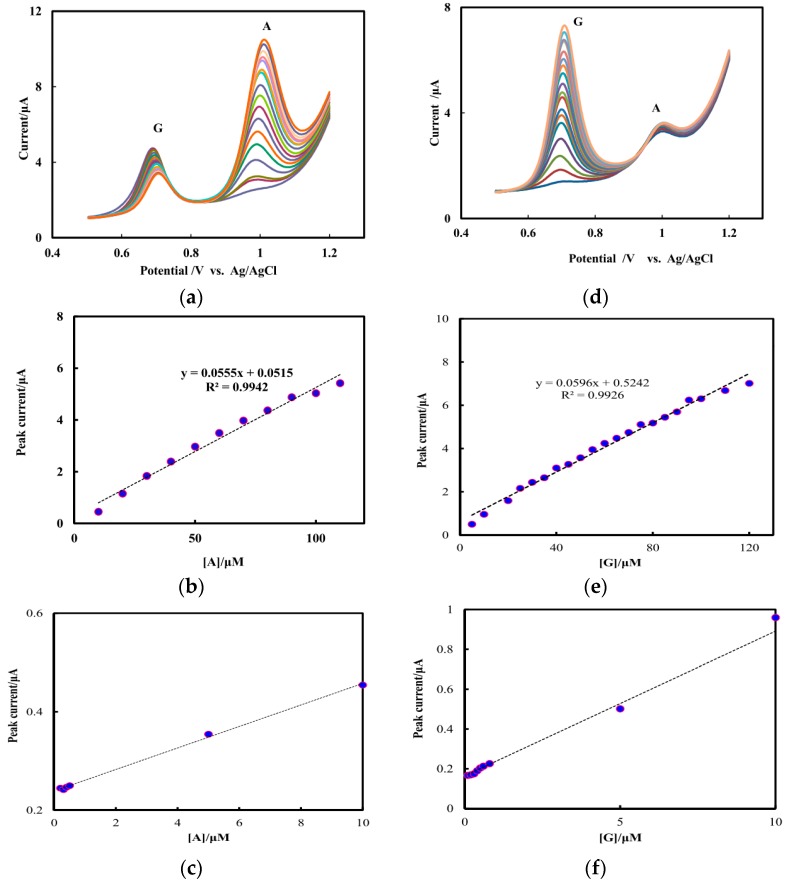
(**a**) DPVs at varying concentrations of A in the presence of G (25 μM); (**b**) Plot of the oxidation peak currents versus different concentrations of A; (**c**) Local enlarged drawing of Figure 4b at low concentrations of A; (**d**) DPVs at varying concentrations of G in the presence of A (25 μM); (**e**) Plot of the oxidation peak currents versus different concentrations of G; (**f**) Local enlarged drawing of Figure 4e at low concentrations of G. The DPVs were operated at a pulse amplitude of 50 mV, pulse width of 50 ms and a scan rate of 50 mV/s, respectively.

The oxidation of G and A in their mixture was investigated by DPV at the NF/CHT-ARGO/GCE, respectively, when the concentration of one species changed, the concentration of the other species remained constant. Figure 4a shows that the oxidation peak current increases linearly with the increasing concentration of A ranging from 0.2 to 110 μM in 50 mM PBS, pH 7.0 containing G (25 μM) (Figure 4b). obtained to be 0.2 μM (Figure 4c). A calibration equation (Equation (1)) for A was then calculated (Figure 4b). Similarly, a linear relationship was found in the range of 0.1–120 μM with a calibration equation (Equation (2)) (Figure 4d,e) for G, in 50 mM PBS, pH 7.0 containing A (25 μM). The detection limit for G was 0.1 μM (Figure 4f). Table 1 shows the comparison of the behaviour of different modified electrodes for the detection of DNA bases.

(1) $$I_{A}(\mu A)=0.0555\;C_{{}_{A}}(\mu M)+0.0515\;(R^{2}=0.994)$$

(2) $$I_{G}(\mu A)=0.0596\;C_{G}(\mu M)+0.5242\;(R^{2}=0.993)$$

### 3.5. Potential Applications of NF/CHT-ARGO/GCE

**Figure 5:**
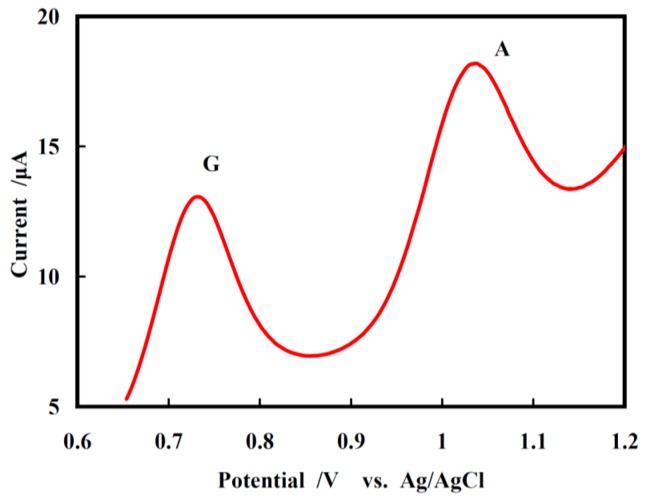
DPVs of G and A in the calf thymus DNA sample in 5 mL, pH 7.0 PBS (phosphate buffer solution) on NF/CHT-ARGO/GCE at a scan rate of 50 mV s^−1^.

The potential applications of the modified GCE were verified in a real DNA. The calf thymus DNA sample was prepared as indicated in Section 2.4. The oxidation peak currents of the DNA sample were clearly shown to be 5.310 μA and 6.000 μA on the NF/CHT-ARGO/GCE by DPV (Figure 5), which were caused by the oxidation of G and A groups, respectively. According to Equations (1) and (2), the concentrations of G and A for calf thymus DNA was calculated to be 80.3 ± 1.0 μM and 107.1 ± 1.0 μM, respectively. So, the ratio of G to A was determined to be 0.75, which was close to the value (0.77) determined by the HPLC method (data not shown).

### 3.6. Reproducibility, Stability and Interferences

To evaluate the reproducibility of the proposed sensor, five NF/CHT-ARGO-modified GCEs were prepared in the same manner, and DPV responses to the mixture containing 20 μM G and 25 μM A in 50 mM PBS (pH 7.0) were investigated. The relative standard deviation (RSD, *n* = 5) were concluded to be 3.45% of G and 4.33% of A, indicating that the reproducibility of the proposed electrode was good. 

To evaluate the long-term stability, the DPV responses to the mixture containing 20 μM G and 25 μM A were detected for 15 days. The RSDs (*n* = 15) were 2.53% of G and 2.87% of A (data not shown). The DPV responses of the prepared electrodes, which were stored at 4 °C, were determined at the interval of three days. The RSDs (*n* = 5) were 2.82% of G and 2.69% of A (data not shown), suggesting that the stability of the proposed electrode was high.

The anti-interference ability of NF/CHT-ARGO/GCE was evaluated by adding possible interferents to samples containing 20 μM G and 25 μM A in 50 mM PBS (pH 7.0). As shown in Table 2, it was found that uric acid, ascorbic acid, vitaminB1, vitaminB2, L-Lysine, Glycine and glucose had little effect on the detection of G or A, indicating that the NF/CHT-ARGO/GCE had a good selectivity and anti-interference ability for measuring G and A. The interference percentages (I%) were calculated using Equation (3):

(3) $$I\%=\left(1-\frac{I_{p}-I_{p0}}{I_{p0}}\right)\times 100\%$$

where, I*_p_* is the oxidation peak current of A or G in the presence of one of the interferences, I_*p*0_ is the oxidation peak current of A or G in the absence of an interference. Obviously, the anti-interference ability of the electrode would be stronger when I% value is close to 100%.

## 4. Conclusions

A new electrochemical sensor based on NF, ARGO and CHT functional membrane-modified glassy carbon electrode was proposed for the simultaneous detection of A and G. CHT may help to disperse ARGO homogeneously in the functional membrane and improve the electrochemical properties of the proposed electrode. The negatively charged NF may adsorb more positively charged guanine and adenine molecules to enhance the oxidation signals. The proposed modified electrode could be applied to quantify G and A with high reproducibility, stability, and sensitivity.

## Figures and Tables

**Table 1:** Comparison of the performances of different chemically modified electrodes for the detection of DNA bases.

Working Electrode	Analyte	Detection Limit (μM)	Linear Range (μM)	Technique	Reference
NF/CHT-ARGO/GCE	A	0.2	0.2–110	DPV	This work
	G	0.1	0.1–120		
GMC/GCE	A	0.63	25-200	DPV	[15]
	G	0.76	25–150		
GS/IL/CHT/GCE	A	0.45	1.5–350	DPV	[17]
	G	0.75	2.5–150		
Graphene-NF/GCE	A	0.75	8–150	DPV	[18]
	G	0.58	4–200		
Nano-ZSM-5/IL	A	9.5	10–300	DPV	[20]
	G	4.8	10–300		
TNFs/GONs/SPCE	A	1.71 × 10^−3^	0.1–10	DPV	[22]
	G	-	-		
MWCNT-Fe_3_O_4_@PDA-Ag/CPE	A	5.66	10–120	DPV	[23]
	G	1.47	8–130		
PANI/MnO_2_/GCE	A	2.9	10–100	DPV	[38]
	G	4.8	10–100		
ZnS-PEDOT-RGO/GC-RDE	A	0.141	0.5–150	DPV	[39]
	G	0.116	0.5–150		
TAN-AgNP-PANT/CPE	A	2.8	1–200	DPV	[40]
	G	4	0.9–140		
Pt–Pd/PSi–CNTPE	A	0.03	0.06–55.0	DPV/AMP	[41]
	G	0.01	0.04–53.4		

GCE: glassy carbon electrode; NF: nafion; CHT: chitosan; GS: graphene sheets; IL: ionic liquid; GMC: graphitized mesoporous carbon; PANI: polyaniline; PEDOT: poly (3,4-ethylenedioxythiophene); GC-RDE: glassy carbon-rotating disk electrode; PSi–CNTPE: mixing of Nujol oil, graphite powder, MWCNTs and polycrystalline Si powder; TNFs: titanium dioxide nanofibers; GONs: graphene oxide nanosheets; SPCE: screen-printed carbon electrode; TAN-AgNP-PANT/CPE: 1,3,5-Trithiane-Ag-nanoparticles decorated polyaniline nanofibers; PDA: polydopamine; MWCNT: multi-walled carbon nanotube; DPV: differential pulse voltammetry; AMP: amperometry.

**Table 2:** Influence of interferences in the determination of G and A.

Signal Change	Uric Acid	Glucose	Ascorbic Acid	Vitamin B1	Vitamin B2	L-Lysine	Glycine
A	101.6%	101.5%	101.8%	104.0%	99.3%	102.3%	103.6%
G	102.2%	97.2%	101.3%	95.9%	96.6%	102.6%	97.3%

The experiments were carried out in 5.0 mL, 50 mM, pH 7.0 PBS containing 20 μM G and 25 μM A, using DPVs of NF/CHT-ARGO/GCE. The concentrations of the interferences were 200 μM.
